# Neferine inhibits vascular smooth muscle cell proliferation and migration in atherosclerosis by targeting CYS130 and PHE252 in PTEN

**DOI:** 10.3389/fphar.2026.1806042

**Published:** 2026-05-28

**Authors:** Qi Jin, Qingqing Liu, Chenghao Ruan, Zhen Xie, Xinzhi Gao, Yanyan Jiang, Fei Jiang, Baoping Xie

**Affiliations:** 1 Key Laboratory of Prevention and Treatment of Cardiovascular and Cerebrovascular Diseases (Gannan Medical University), Ministry of Education, Ganzhou, Jiangxi, China; 2 Jiangxi Provincial Key Laboratory of Tissue Engineering, Ganzhou, Jiangxi, China; 3 College of Rehabilitation, Gannan Medical University, Ganzhou, Jiangxi, China; 4 The Affiliated Traditional Chinese Medicine Hospital, Guangzhou Medical University, Guangzhou, Guangdong, China

**Keywords:** alkaloid, atherosclerosis, neferine, *Nelumbo nucifera*, vascular smooth muscle cell

## Abstract

**Introduction:**

The abnormal proliferation and migration of vascular smooth muscle cells (VSMCs) are significant pathological factors contributing to atherosclerosis (AS). Neferine (Nef) is a dibenzylisoquinoline alkaloid isolated from the *Nelumbo nucifera*. Our previous studies revealed that Nef has a good pharmacological effect on AS by inhibiting macrophage glycolytic reprogramming. However, the regulatory mechanism of Nef on abnormal VSMC proliferation and migration, specifically the direct target, has not been clarified.

**Method:**

High-fat diet (HFD) induces AS model and uses different concentrations of Nef treatment, oil red staining, EdU and scratch experiments to evaluate the pharmacological effects of Nef, molecular docking, molecular dynamics simulation, CETSA, DART experiments to elucidate the direct target of Nef, Western Blot, immunofluorescence and immunohistochemistry to detect the effects of Nef on PTEN/AKT pathways and complexes.

**Result:**

Nef exhibited significant pharmacological effects against AS, markedly inhibited the abnormal proliferation and migration of VSMCs, reduced the expression levels of PCNA and p-AKT, and promoted PTEN expression. Furthermore, competitive blockade of PTEN by Bpv (HOpic) substantially diminished the regulatory effects of Nef on VSMC proliferation and migration. Mechanistically, we identified PTEN as a direct target of Nef and CYS130 and PHE252 were identified as the binding sites of the Nef-PTEN complex. Molecular dynamics simulations, co-immunoprecipitation (Co-IP) experiments demonstrated that Nef targeted PTEN to promote the formation of the PTEN/AKT complex and inhibit AKT phosphorylation. Competitive PTEN inhibition markedly reduced the regulatory effect of Nef on lipid levels and formation.

**Conclusion:**

This study presents a novel therapeutic drug of Nef for AS by targeting the PTEN-AKT complex and PTEN.

## Graphical Abstract

**Figure:**
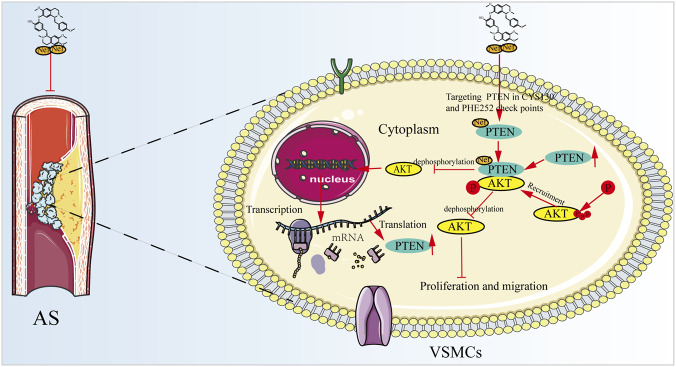
Neferine targets PTEN in combination with CYS130 and PHE252 check points to promote the formation of PTEN/AKT complexes and inhibit AKT phosphorylation, thereby inhibiting the proliferation and migration of vascular smooth muscle cells in atherosclerosis.

## 1 Introduction

Atherosclerosis (AS) is a chronic inflammatory disease characterized by abnormal lipid metabolism and the accumulation of atherosclerotic plaques (Libby, 2021). It is the pathological basis of cardiovascular diseases, including myocardial infarction, angina pectoris, and heart failure (Xie et al., 2025d). The pathogenesis of AS is very complex, involving macrophage foaming, abnormal proliferation and migration of vascular smooth muscle cells (VSMCs), oxidative stress injury of endothelial cells, and infiltration of inflammatory factors (Xie et al., 2025d). Among them, abnormal proliferation and migration of VSMCs are regarded as the main mechanisms of AS formation. Abnormally proliferating VSMCs and foamy macrophages form the core area of AS plaques (Miano et al., 2021; Durham et al., 2018). Therefore, inhibiting the abnormal proliferation and migration of VSMCs is often used as a new strategy for anti-AS and as a classic model for drug screening (Li et al., 2025; Lin et al., 2021). Moreover, during AS formation, VSMCs under go a phenotypic transition from contractile to synthetic, and the expression of contraction-related proteins, including α-smooth muscle actin and troponin (calponin), is significantly reduced (Chakraborty et al., 2023).

Phosphatase and tensin homolog (PTEN) is a member of the tyrosine kinase family that regulates post-translational modifications of target proteins, including dephosphorylation, acetylation, and ubiquitination (Haddadi et al., 2020). As a tumor suppressor, PTEN is frequently mutated or deleted in cancer and regulates glucose metabolism through the PI3K-AKT pathway (Chen et al., 2018; Qian et al., 2019). Recently, an increasing number of studies have revealed that PTEN is vital for VSMC proliferation and migration, specifically in traditional Chinese medicine (TCM) and natural products (Zhu et al., 2019). Dipsacoside B inhibits VSMC migration and proliferation and blunts neointimal formation by upregulating PTEN expression (Quan et al., 2022). Bushen Huoxue formula alleviates vascular calcification in rats with chronic kidney disease by inhibiting the PTEN/PI3K/AKT signaling pathway (Guo et al., 2025; Quan et al., 2022). Neferine (Nef) is a dibenzylisoquinoline alkaloid extracted from *Nelumbo nucifera*, which exhibits pharmacological effects, including anti-cancer, anti-depression, anti-oxidation, anti-arrhythmic, anti-thrombotic, anti-inflammatory, and anti-AS (Marthandam et al., 2018). Our previous study confirmed that dibenzylisoquinoline alkaloids can significantly inhibit the abnormal proliferation and migration of VSMCs (Feng et al., 2023). Moreover, Nef targets eEF1A1 to regulate the glycolytic reprogramming of macrophages and inhibit the atherosclerotic plaque formation (Xie et al., 2025b). However, the direct targets and mechanisms by which Nef inhibits VSMC proliferation and migration have not yet been clarified. In this study, we confirmed that Nef has a good anti-AS pharmacological effect and that PTEN may be a direct target of Nef, inhibiting VSMC proliferation by a non-modified small-molecule target identification method. Furthermore, we investigated the mechanism by which Nef inhibits the proliferation and migration of VSMCs.

## 2 Materials and methods

### 2.1 Animal experiment and drug administration

In total, eight male wild-type C57BL/6J mice and 49 male ApoE^−/−^ mice (25 ± 2 g) between 6–8 weeks of age were purchased from JiangSu Huachuang Sino Laboratory Animal company (Suzhou, China, No: SCXK (su)-20200,009), and maintained in specific pathogen-free (SPF) conditions, including temperature-controlled (23 °C ± 2 °C), humidity-controlled (55% ± 15%) on a 12 h light/dark cycle with free access to food and water in independent ventilation cages (IVC). The feed, water and bedding were sterilized through high-temperature and high-pressure treatment (Xie et al., 2025a). The animal experiment protocols of this study strictly adhered to ethical standards and national guidelines for animal care and use, and were approved by the Animal Experimentation Ethics Committee of Gannan Medical University (No.2025524). After 1 week adaptation, the ApoE^−/−^ mice were randomly divided into the Model group (n = 7), Nef (10 and 20 mg/kg/day) group (n = 7), Model + BpV (200 μg/kg/day) group (n = 7) and Nef (20 mg/kg/day) + BpV (200 μg/kg/day) group (n = 7). All ApoE^−/−^ mice were fed a high-fat diet (HFD), while the normal-chow-fed wild-type C57BL/6 mice served as the control mice (Xie et al., 2025b). Nef suspension was prepared at a corresponding concentration using 0.9% physiological saline, and then ultrasonicated for 30 min. Mice in the Nef group were intragastrically administered the corresponding dose of the drug. Mice in the Model + BpV (200 μg/kg/day) group and Nef (20 mg/kg/day) + BpV (200 μg/kg/day) group received intraperitoneal injections with the corresponding dose of the drug, while the model group and control group mice were intragastrically administered or intraperitoneally injected with the same volume of 0.9% physiological saline. After 16 weeks of treatment, the mice were anesthetized by isoflurane (R510-22–10, RWD, China) prior to sacrifice, and the abdominal aorta, aortic arch and serum were collected for subsequent experiments.

### 2.2 Oil Red O staining of arcus aortae, aortaventralis and aortic valves

The Oil Red O Staining of arcus aortae, aortaventralis and aortic valves was performed according to our previous research (Xie et al., 2025c). Briefly, the tissues were processed using frozen sectioning techniques, and fixed with 4% paraformaldehyde (BL539A, Biosharp, China) for 24 h. Then, the atherosclerotic lesions in aortaventralis, arcus aortae and aortic valves were assessed using Oil Red O staining (Cat: O0625, Sigma, USA) according to the instructions of the test kit and our previous research (Xie et al., 2025c). Following staining, the tissues or slices were washed 3 times with phosphate-buffered saline (PBS, Solarbio, Beijing, China), and then incubated with 60% isopropanol for 1 min. Images of atherosclerotic lesions were captured using a microscope, and the atherosclerotic lesion areas were quantified as the percentage of Oil Red O-positive area relative to the total staining area respectively, using ImageJ analysis software, as in our previous study (Xie et al., 2025c).

### 2.3 Biochemical analysis

To evaluate the regulation of Nef on the lipid levels in mice induced by HFD, the kits for total cholesterol (TC), triglycerides (TG), low-density lipoprotein cholesterol (LDL), high-density lipoprotein cholesterol (HDL) were purchased from Jiancheng Bioengineering Institute (Nanjing, China). Detection methods were measured according to the manufacturer’s instructions and our previous study (Xie et al., 2025c).

### 2.4 Cells culture and treatments

Mouse aortic vascular smooth muscle cells (MOVAS) were obtained from the American Type Culture Collection (ATCC, VA, USA). The cells were cultured in Dulbecco’s Modified Eagle Medium (DMEM, Gibco, USA) at 37 °C and 5% CO_2_. MOVAS cells were seeded in 12-well plates or 6-well plates at a density of 1 × 10^5^ cells/well. After 4–6 h of starvation, they were exposed to 3% FBS for 48 h with or without Nef (0.5 µM, 1 μM, 2 μM), as well as BpV (HOpic) (BpV, HY128693, MedChemExpress, Shanghai, China), which is a selective PTEN antagonist. Then, MOVAS cells were used for subsequent testing.

### 2.5 Cells viability and proliferation

MOVAS cells were seeded in 96-well plates at a density of 1 × 10^4^ cells/well. Cells culture and treatments were consistent with “2.4”. Then, the Cell Counting Kit-8 (CCK-8, CA1210, Solarbio, China) reagent was used to evaluate cell viability and proliferation. 10 μL of CCK-8 solution was added to each well. After a 1h incubation period, the absorbance optical density (OD) at 450 nm was measured by a microplate reader (Varioskan LUX, USA) (Xie et al., 2025b).

### 2.6 EdU staining

For the EdU staining cell proliferation assay, after 48 h of drug treatment, culture medium containing 50 μM EdU was added to each group, and the cells were incubated for 2 h at 37 °C. EdU staining was then conducted using BeyoClick™ EdU-488 imaging kit (Beyotime, C0071S, China) according to the manufacturer’s protocol and our previous research (Feng et al., 2023). Images were recorded by a fluorescence microscope (Leica, DNI3000, German). As in our previous studies, the rate of EdU-positive cells was calculated by ImageJ, and the rate of EdU-positive cells = total number of EdU-positive cells/total number of cells (Feng et al., 2023).

### 2.7 Quantitative real-time polymerase chain reaction (qRT-PCR)

After 48 h of drug treatment, total RNA was extracted from MOVAS cells by Trizol reagent (Qiagen, Frederick, MD, USA) and cDNA was synthesized by a reverse transcription kit (CW3371M, CWBIO, Taizhou, China) according to the manufacturer’s instructions. The cDNA was stored at −20 °C. Primers for PTEN, AKT, PCNA and GAPDH were designed and purchased from Sangon Biotech (Shanghai, China) (Table 1). Next, qPCR was performed on a Real-Time qPCR Thermal Cycler (Applied Biosystems, USA) using the SYBR Green method (CW3390M, CWBIO, Taizhou, China) according to the manufacturer’s instructions. The data were analyzed by the ^ΔΔ^Ct method following our previous studies (Xie et al., 2025b), and GAPDH was used as an internal control.

**TABLE 1:** qRT-PCR primer sequence.

qRT-PCR primer sequence	Forward	Reverse
PTEN	5′-CAC​AGA​ATT​CCA​GAC​ATG​ACA​GCC​ATC​ATC-3′	5′-GTG​GAT​CCT​CTA​GGT​TTA​TCC​CTC​TTG-3′
AKT	5′-CAG​CCA​GAC​CTC​GTT​CCT​CTT​AG-3′	5′-CAA​TGC​AGA​GGG​GTG​CAG​G-3′
PCNA	5′-CAG​CCA​GAC​CTC​GTT​CCT​CTT​AG-3′	5′-GAG​CCT​CCA​GCA​CCT​TCT​TCA​G-3′
GAPDH	5′-CTC​ATG​ACC​ACA​GTC​CAT​GC-3′	5′-TTC​AGC​TCT​GGG​ATG​ACC​TT-3

### 2.8 Wound-healing assay

The wound-healing assay was used to assess the cell migration. MOVAS cells were cultured in 12-well plates at a density of 1 × 10^5^ per well. As described in previous studies, scratch lines were created with a 200 μL pipette tip, and images of the scratches were captured at 0 h by a microscope. Then, cell culture and treatments were consistent with “2.4″, and the scratch lines were observed with a microscope after 24 h of incubation (Xiao et al., 2023). The area of the original and final scratch lines were measured using ImageJ software.

### 2.9 Western blot

After 48 h of drug treatment, total protein was extracted from MOVAS cells using RIPA lysis buffer (G2002-100ML, Wuhan Servicebio, China) supplemented with 1 mM PMSF (P0100, Solarbio, China) at 4 °C for 30 min, and the protein lysate was obtained after centrifugation (4 °C, 12,000 rpm, 10min). Protein concentrations were determined using the Bradford assay (PC0020, Solarbio, China). Samples were prepared by adding 5× loading buffer and boiling at 100 °C for 5 min, Then, equal amounts of protein were separated by 10% sodium dodecyl sulfate-polyacrylamide gel electrophoresis (SDS-PAGE) and subsequently transferred onto polyvinylidene difluoride (PVDF) membranes. Subsequently, the membranes were blocked with 5% bovine serum albumin (BSA) for 1 h and then incubated overnight at 4 °C with the following primary antibodies: anti-PCNA (GB11010-50, Servicebio, China), anti-AKT and p-AKT (bsm052010R and bs-10134R, Bioss, Beijing, China), anti-PTEN (PTM-5119, PTM BIO, Huangzhou, China), anti-β-actin (Cat: AC038, Abclonal, China). Next, the PVDF membranes were incubated with the horseradish peroxidase (HRP)-labeled goat anti-rabbit IgG secondary antibody (A0208, Beyotime, China) at room temperature for 1 h, and cleaned 3 times with TBST. The protein bands were stained using enhanced chemiluminescence (ECL) reagent (G3308, GBCBIO, China). β-Actin was used as an internal loading control, and the optical densities of the bands were quantified using ImageJ analysis software.

### 2.10 Co-immunoprecipitation (CoIP)

MOVAS cells were seeded on 25 cm^2^ culture dishes and divided into Control group, Model group, and Nef (2 μM) group with or without BpV. MOVAS cells processing and administration were performed as described in “2.4”. After 48 h of drug treatment, proteins were extracted using RIPA lysis buffer supplemented with 1 mM PMSF at 4 °C for 30 min. Protein concentration was determined by the Bradford method, and equal amounts of protein were taken respectively. Next, 1 mg of PTEN antibody was added to the sample and incubated for 1 h at room temperature. After that, 40 μL of agarose beads (PR40025, Proteintech, China) were added to each sample and incubated overnight at 4 °C. After centrifugation at 3500 rpm for 5 min, the supernatant was discarded, and the agarose beads were washed 5 times with PBS for 5 min, and then boiled in loading buffer at 100 °C with for 7 min. Western blot analysis was performed on the CoIP sample.

### 2.11 Cellular thermal shift assay (CETSA)-WB

CETSA-WB experiment was conducted as previously described (Xie et al., 2025b). Briefly, after 48 h of drug treatment, proteins were extracted using RIPA lysis buffer supplemented with 1 mM PMSF at 4 °C for 30 min. Protein concentration was determined by the Bradford method. The protein lysate was aliquoted into PCR tubes, and treated with Nef (20 μM) or DMSO at room temperature for 1 h in horizontal shaker. Then, the protein lysate was divided into 6 portions and heated at specified temperatures (42 °C, 47 °C, 52 °C, 57 °C, 62 °C, 67 °C) for 5 min. Subsequently, it was cooled at 4 °C for 3 min, and subjected to 10 min of centrifugation (12,000 rpm, 4 °C). Then, the soluble supernatant was boiled in loading buffer at 100 °C for 7 min, and detected by WB detection analysis in 10% SDS-PAGE gels.

### 2.12 Drug affinity responsive target stability (DARTS)-WB

DARTs-WB experiment was conducted as previously described (Xie et al., 2025b). Briefly, protein lysate of MOVAS cells was obtained from ice-cold M-PER lysis buffer (Thermo Fisher Scientific Inc., USA) supplemented with PMSF (1 mM) and protein phosphatase inhibitors (1 mM). Then, the protein lysate was diluted with M-PER lysis buffer to a final protein concentration of 6 mg/mL. The protein lysate was mixed with 1×TNC buffer, divided into 5 tubes, and incubated at room temperature with DMSO or Nef (0, 2.5, 5 and 10 μM) for 1 h (Xie et al., 2025b). Following incubation, each sample was split into 5 μL aliquots (50 μg of proteins) with pronase (Sigma, 10,165,921,001, USA) for 10 min at room temperature. Digestion was further stopped by adding 5× loading buffer, boiling at 100 °C for 5 min, and loading onto 10% SDS-PAGE gels for WB (Xie et al., 2025c).

### 2.13 Plasmid transfection

PcDNA-PTEN, pcDNA-vectors and pcDNA-PTEN-MU were designed and synthesized by FENGHUISHENGWU (Changsha, China). In order to obtain large quantities of plasmids for the CETSA and DARTs experiments, the plasmids were transformed into *Escherichia coli*, and plasmid extraction was performed following the instructions of the plasmid extraction kit (TianGen, Beijing, China) and our previous study (Xie et al., 2025c). Then, the plasmids were transfected into 293T cells using Lipofectamine 3000 (L3000015, Thermo Fisher, USA) following the manufacturer’s instructions. After 24 h of transfection, the cells and proteins were used for subsequent drug administration.

### 2.14 Molecular docking and computational method

The receptors were obtained from the PDB (PTEN: 6ZMO, AKT: 7NH5) in PDB format for docking purposes. These proteins were prepared by removing water, adding hydrogen, performing energy minimization, and applying a series of processing steps. Then, autodock Vina was used for molecular docking and visualization. Finally, the center coordinates and box were selected to dock the ligand, and the best position of Nef was selected by the docking score (Wang et al., 2023). For PTEN and AKT protein-protein interactions, H-DOCK was used to predict the protein-protein binding complex (Yan et al., 2020). Molecular dynamics (MD) simulations were performed for ligand-protein and protein-protein complex structures to evaluate the binding pose, and the most suitable ligand-protein and protein-protein structures were used for further analysis (Roe and Cheatham, 2013). MD simulations were conducted using AMBER (version 20), and the protein-protein and protein-ligand complexes were subjected to 100 ns MD simulations. The binding free energy of the protein-ligand complexes was calculated using MMGBSA (Wang et al., 2019).

### 2.15 Immunohistochemical analysis

Arcus aortae slices were washed three times with PBS and incubated with 0.1 mL Triton X-100 (0.5%) for 14 min. The slices were blocked with 5% BSA (A8020, Solaibio, China) at room temperature for 30 min. The slices were then incubated with anti-PCNA (GB11010-100, Servicebio, Wuhan, China) at 4 °C for 8–10 h or overnight, washed three times with PBS, and incubated with goat anti-rabbit secondary antibodies (GB23204, Servicebio, Wuhan, China) for 1 h at room temperature. After three times washing with PBS, the nuclei were stained using 4,6-diamidino-2-phenylindole (DAPI, GeneCopoeia) for 2 min and washed three times with PBS. These stained slices were captured as images using a microscope (SLIDEVIEW VS200, Olympus), and the positive area of PCNA was analyzed using ImageJ software.

### 2.16 Immunofluorescence assay

MOVAS cells were washed three times with PBS, and fixed with 4% paraformaldehyde (BL539A, Biosharp, China) for 20 min at room temperature. Then, the cells were incubated with 0.1 mL Triton X-100 (0.5%, P0096, Beyotime, China) for 14 min, and blocked with 5% BSA for 30 min at room temperature. Next, the cells were incubated with anti-PTEN at 4 °C for 8–10 h or overnight, washed with PBS for three times, and then incubated with fluorescein-conjugated goat anti-rabbit secondary antibodies (A0423, Beyotime, China) for 1 h, followed by three washes with PBS. Nuclei were stained using DAPI (P0131, Beyotime, China) for 2 min and washed thrice with PBS. Immunofluorescence images of each treatment group were obtained using a fluorescence microscope (Leica, USA) and analyzed using ImageJ software.

### 2.17 Statistical analysis

All experiments in this study were repeated four times (n = 4–6), and all data were presented as mean ± standard deviation and were normalized. Statistical analysis was performed using SPSS 20, and graphing and visualization were conducted using GraphPad Prism 8.0 software (La Jolla, CA, USA). One-way analysis of variance (ANOVA) was used for inter-group comparisons, and differences between two groups were assessed using an unpaired Student’s t-test. *P* value less than 0.05 (*P* < 0.05) was considered statistically significant.

## 3 Results

### 3.1 Nef inhibits the proliferation of VSMCs and the formation of atherosclerotic plaques

**FIGURE 1:**
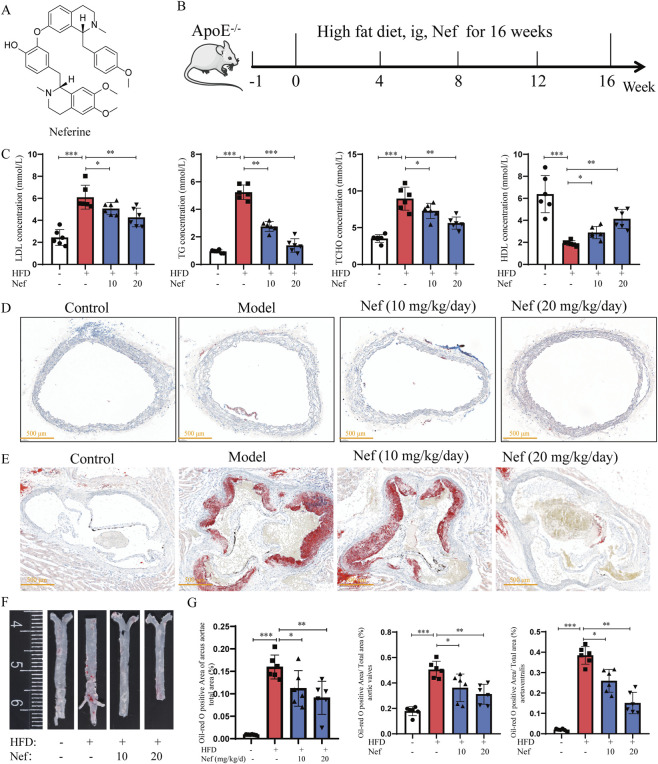
Nef significantly reduces AS plaque formation induced by HFD. **(A)** Structural formula of Nef. **(B)** Schematic diagram of the animal experiment. **(C)** Results of the blood lipid biochemical tests (n = 6). **(D–F)** Results of Oil Red O staining of the aortic arch, aortic valve, and aortaventralis (n = 6). **(G)** Statistical analysis of Oil Red staining of the aortic arch, aortic valve, and aortaventralis (n = 6). ^*^ *P* < 0.05, ^**^ *P* < 0.01, ^***^ *P* < 0.001 versus model group, scale column:100 μm.

To study the anti-atherosclerotic pharmacological effects of Nef, we established an AS model in ApoE ^−/−^ mice by feeding them an HFD and administering Nef at different concentrations via gavage. Nef is a dibenzylisoquinoline alkaloid extracted from *Nelumbo nucifera* (Figure 1A). After 16 weeks of treatment, the model was evaluated by quantifying of serum T-CHO, TG, LDL-C, and HDL-C levels (Figure 1B). As shown in Figure 1C, compared with the control group, the results demonstrated a significant increase in T-CHO, TG, and LDL-C levels and a decrease in HDL-C content in the model group (all *P* < 0.05). However, Nef (10 and 20 mg/kg/day) significantly reduced serum T-CHO, TG, and LDL levels and increased HDL levels in a dose-dependent manner compared to the model group (Figure 1C). Atherosclerotic plaques usually occur in the aortic arch, aortic valve, and abdominal aorta, affecting blood and oxygen supply to the heart. Oil Red O staining is the most classic method for evaluating atherosclerotic plaques (Wen et al., 2024; Xue et al., 2025). Thus, atherosclerotic lesions in the aortic arch, aortaventralis, and aortic valve were assessed using Oil Red O staining. More importantly, the model group revealed larger atherosclerotic lesion areas in the aortic arch, aortaventralis, and aortic valve regions compared to the control mice. However, Nef (10 and 20 mg/kg/day) treatment at both experimental doses resulted in alleviated atherosclerotic lesion areas compared to the model group, including in the aortaventralis, arcus aortae, and aortic valves (Figures 1D–G). Consequently, our results indicate that Nef at the experimental dose has good anti-atherosclerotic pharmacological effects, significantly inhibits AS plaque formation, and reduces blood lipid levels in the serum.

### 3.2 Nef inhibits the proliferation and migration of MOVAS

**FIGURE 2:**
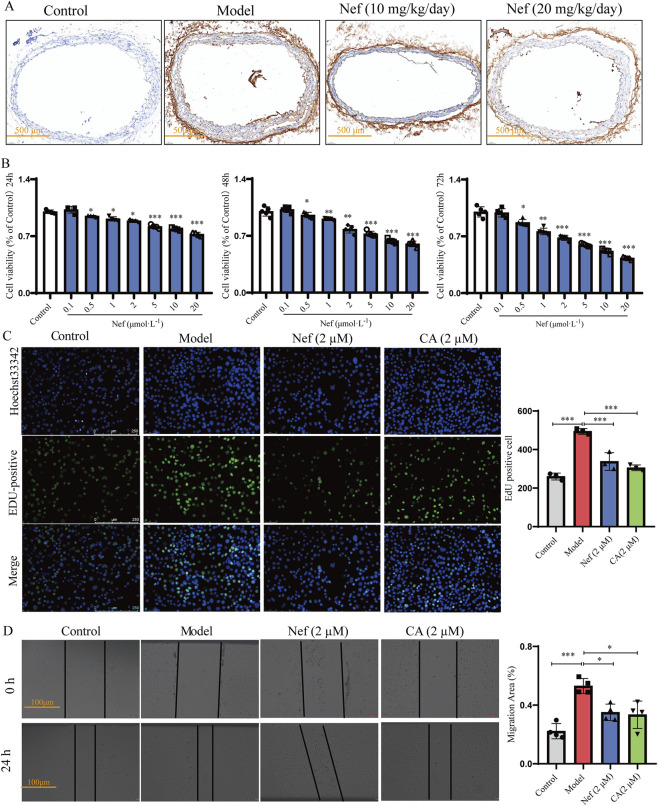
Nef significantly inhibited the proliferation and migration of MOVAS. **(A)** Immunohistochemical analysis of PCNA in aortic arch (n = 6). **(B)** The CCK-8 was used to assess the effect of Nef on VSMC proliferation (n = 5). **(C)** EdU staining was used to assess the effect of Nef on MOVAS proliferation (n = 4). **(D)** Scratch assay was used to detect the effect of Nef on MOVAS migration (n = 4). *P < 0.05, **P < 0.01, ***P < 0.001 versus model group, scale column:100 μm.

Abnormal VSMC proliferation and migration are generally considered to be the main pathogenesis of AS (Basatemur et al., 2019). MOVAS are the most common type of VSMCs and are frequently utilized in studies on abnormal proliferation, migration of VSMCs, and atherosclerosis (Xiao et al., 2023; Xiang et al., 2024). We previously isolated 13 dibenzylisoquinoline alkaloids from *Nelumbo nucifera* that significantly inhibited VSMC proliferation and migration (Feng et al., 2023). Immunohistochemical analysis revealed that Nef treatment significantly decreased PCNA expression in the aortic arch, which was correlated with the abnormal proliferation and migration of MOVAS (Figure 2A). However, the specific mechanisms underlying this phenomenon remain unclear. Here, we first examined the effect of Nef on MOVAS proliferation. As presented in Figure 2A, compared with the control group, the expression of PCNA in the model group was significantly increased, while Nef exhibited dose-dependent inhibition of PCNA expression compared to the model group. The cell activity and proliferation assays showed similar results: Nef inhibited MOVAS proliferation in a dose-dependent manner compared to the control group, and the Nef treatment for 48 h showed a significant inhibitory effect on MOVAS cell proliferation (Figure 2B). Abnormal proliferation and migration of VSMCs and foamed macrophages are primary contributors to AS plaque formation (Xue et al., 2022). Consistent with the literature, we used a 3% fetal bovine serum (FBS) concentration to stimulate abnormal VSMC proliferation and migration (Xiao et al., 2023), and assessed the effect of Nef on MOVAS proliferation stimulated by 3% FBS via EdU staining. Nef (2 μM) significantly inhibited MOVAS proliferation induced by 3% FBS, and its inhibitory efficiency was comparable to that of the positive control drug cinnamaldehyde (Figure 2C). In the migration experiment, after 48 h of Nef treatment, Nef significantly inhibited the abnormal migration of MOVAS induced by 3% FBS (Figure 2D). Consequently, our study revealed that Nef reduced AS plaque formation by inhibiting abnormal VSMC proliferation and migration.

### 3.3 Nef directly targets PTEN at Lys64 and Lys154

**FIGURE 3:**
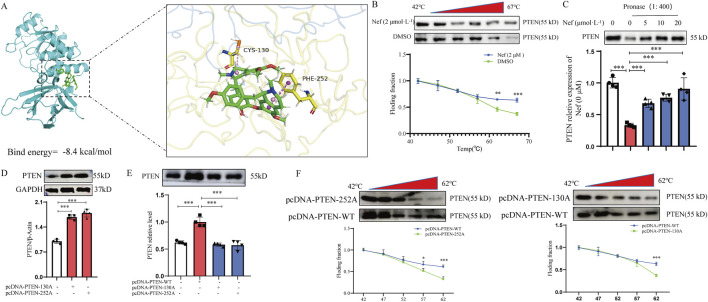
PTEN is a direct target of Nef, and CYS-130 and PHE-252 are the binding sites of the Nef-PTEN complex. **(A)** Molecular docking of the Nef-PTEN complex. **(B)** CETSA experiment of the Nef-PTEN complex (n = 4). **(C)** DARTs experiment of the Nef-PTEN complex (n = 4). **(D)** Mutation plasmid transfection (n = 4). **(E)** CETSA experiment of the Nef-PTEN complex after transfection mutation plasmid (n = 4). **(F)** DARTs experiment of the Nef-PTEN complex after transfection mutation plasmid (n = 4). *P < 0.05, **P < 0.01, ***P < 0.001 versus Nef (0 μM) group or pcDNA-PTEN-WT group.

Increasing evidence suggests that PTEN is vital for the proliferation and migration of VSMCs, and that inhibition of PTEN expression can significantly suppress the proliferation and migration of VSMCs or tumor cells (Coronel-Hernandez et al., 2019; Kadri et al., 2022). To elucidate the molecular mechanisms and specific targets through which Nef inhibits the aberrant proliferation and migration of VSMCs, we performed molecular docking analyses. These analyses revealed that Nef forms a stable Nef-PTEN complex with PTEN, with a binding energy of −8.4 kcal/mol (Figure 3A). We observed that PTEN is a direct target of Nef using CETSA and DARTs experiments. Interestingly, Nef enhanced the thermal stability of PTEN and inhibited its proteolytic degradation by pronase compared with the DMSO group (Figures 3B,C). Moreover, to elucidate the binding site of the Nef-PTEN complex, we designed and synthesized plasmids containing CYS130 and PHE252 mutations based on molecular docking results, which were subsequently transfected into 293T cells (Figure 3D). Then, we observed that the mutation of CYS130 and PHE252 to alanine significantly diminished the thermal and enzymatic stability of the Nef-PTEN complex by CETSA and DARTs experiments (Figures 3E,F). Overall, our research confirmed that PTEN is a direct target of Nef and that CYS130 and PHE252 are the binding sites of the Nef-PTEN complex.

### 3.4 Nef inhibited MOVAS cell proliferation and migration by targeting PTEN

**FIGURE 4:**
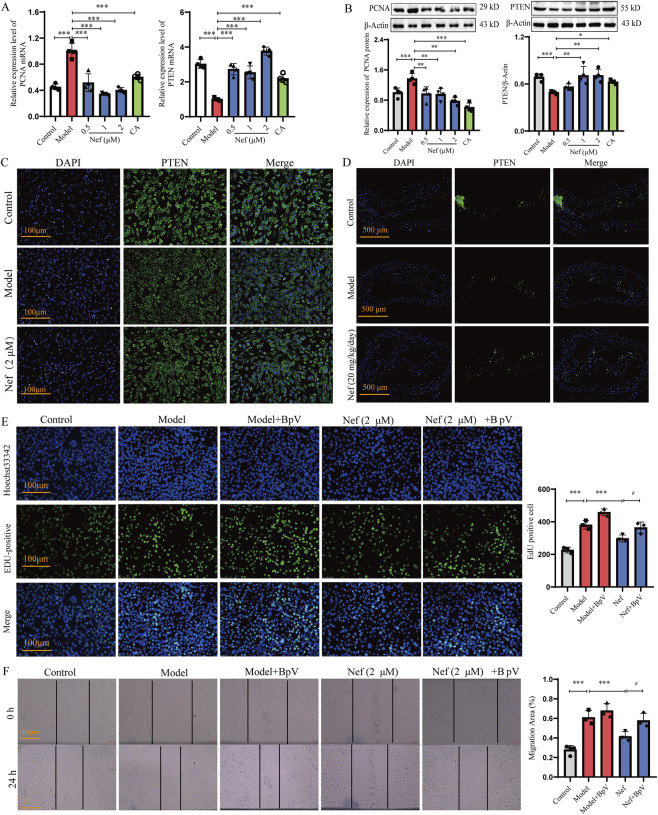
Nef inhibited MOVAS proliferation and migration by targeting PTEN. **(A,B)** The expression of PNCA and PTEN was detected by qRT-PCR and Western blotting (n = 4). **(C)** Immunofluorescence detection of Nef expression on PTEN *in vitro* (n = 4). **(D)** Immunofluorescence assay of PTEN expression in the aortic arch (n = 4). **(E,F)** Competitive antagonism of PTEN significantly reduced the inhibitory effect of Nef on the proliferation and migration of MOVAS cell (n = 4). ^*^ *P* < 0.05, ^**^ *P* < 0.01, ^***^ *P* < 0.001 versus model group, scale column:100 μm.

PTEN, a tumor suppressor gene, is regarded as a biomarker for cell proliferation. Studies have revealed that low PTEN expression usually leads to poor prognosis of tumors and promotes the proliferation and invasion of tumor cells (Tian et al., 2022; Zhang et al., 2022). Accordingly, we examined the effect of Nef on the PTEN/AKT signaling pathway in MOVAS. Our study revealed that Nef significantly inhibited the expression of PCNA mRNA and promoted PTEN expression at both mRNA and protein levels (Figures 4A,B). Then, we used immunofluorescence to examine the effect of Nef on the expression of PTEN both *in vitro* and *in vivo*. Consistent with qPCR and Western blot tests, immunofluorescence assays also confirmed that Nef significantly promoted the PTEN expression in MOVAS induced by 3% FBS (Figure 4C). Notably, compared with the model group, Nef treatment significantly increased PTEN expression in the aortic arch, as depicted by immunofluorescence (Figure 4D). Furthermore, we used the PTEN-specific inhibitor BpV to counteract the biological functions of PTEN and examined the proliferation and migration of MOVAS. PTEN inhibition significantly weakened the regulatory effect of Nef on the abnormal proliferation and migration of MOVAS (Figures 4E,F). Taken together, we observed that Nef targets PTEN to inhibit the abnormal proliferation and migration of MOVAS induced by 3% FBS, and that PTEN is a potential target for anti-atherosclerotic therapies.

### 3.5 Nef inhibits the proliferation and migration of MOVAS by targeting PTEN/AKT complex

**FIGURE 5:**
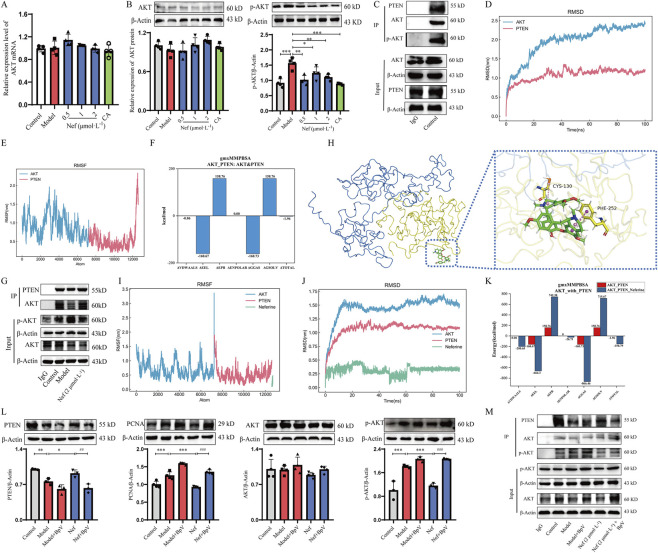
Nef inhibits the proliferation and migration of MOVAS by targeting PTEN/AKT complex. **(A)** Detection of AKT expression by qRT-PCR (n = 4). **(B)** The AKT and p-AKT expression were detected by Western blotting (n = 4). **(C)** Co-IP analysis revealed that PTEN formed a complex with AKT. **(D,E)** RMSD and RMSF of PTEN-AKT complex. **(F)** Binding energy of PTEN-AKT complex. **(G)** Co-IP analysis revealed that Nef promotes PTEN/AKT complex formation. **(H)** Molecular dynamics simulations elucidated the effect of Nef on the PTEN/AKT complex. **(I–K)** RMSD, RMSF, and binding energy of the Nef-PTEN-AKT complex. **(L)** PTEN-specific inhibitor BpV significantly inhibited the regulation of Nef on the expression of PCNA and p-AKT (n = 4). **(M)** PTEN-specific inhibitor BpV significantly inhibited the regulation of the PTEN-AKT complex by Nef. ^*^ *P* < 0.05, ^**^ *P* < 0.01, ^***^ *P* < 0.001 versus model group, ^#^ *P* < 0.05, ^##^ *P* < 0.01, ^###^ *P* < 0.001 versus Nef (2 μM) group, scale column:100 μm (n = 4).

PTEN is the upstream of the AKT signaling pathway. A previous study has identified that PTEN can inhibit cell proliferation and migration by inhibiting PI3K/AKT and AKT/mTOR pathways (Zhen et al., 2020; Xu et al., 2021). Previous studies have observed that PTEN may be a dephosphorylating enzyme involved in the post-translational modification of proteins (Chen et al., 2020), and that increased PTEN expression significantly inhibits the expression of p-AKT (Wu et al., 2021; Hlozkova et al., 2022). We observed that Nef did not affect the expression levels of AKT mRNA or protein but significantly inhibited p-AKT expression (Figures 5B). Our research confirmed that PTEN interacts with AKT to form a PTEN-AKT complex, as demonstrated by Co-IP experiments (Figure 5C). Protein docking and molecular dynamics simulations revealed that PTEN and AKT can form a stable complex (Figures 5D–F). As revealed in Figure 5D, PTEN and AKT can form a stable PTEN-AKT complex at 60 ns and form a relatively stable bond. Notably, we observed that Nef significantly enhanced the PTEN-AKT complex formation (Figure 5G), suggesting that Nef inhibits AKT phosphorylation by promoting the formation of the PTEN/AKT complex. Interestingly, we found that Nef can form a stable complex with PTEN but does not bind to AKT through molecular dynamics simulations (Figure 5H). Consistent with our experimental results, Nef increased the stability of the PTEN-AKT complex compared to the PTEN-AKT complex (Figure 5G), and the 3-phase system of Nef-PTEN-AKT formed the complex more rapidly (Figures 5I,J). Overall, PTEN is a direct target of Nef, and Nef can stably bind to PTEN, promoting PTEN/AKT complex formation and reducing AKT phosphorylation. More importantly, the PTEN inhibitor BpV does not affect the regulation of AKT by Nef. Therefore, our study indicates that Nef inhibits the aberrant proliferation and migration of MOVAS by upregulating PTEN expression and inhibiting AKT phosphorylation.

### 3.6 Nef inhibit atherosclerosis plaque by targeting PTEN

**FIGURE 6:**
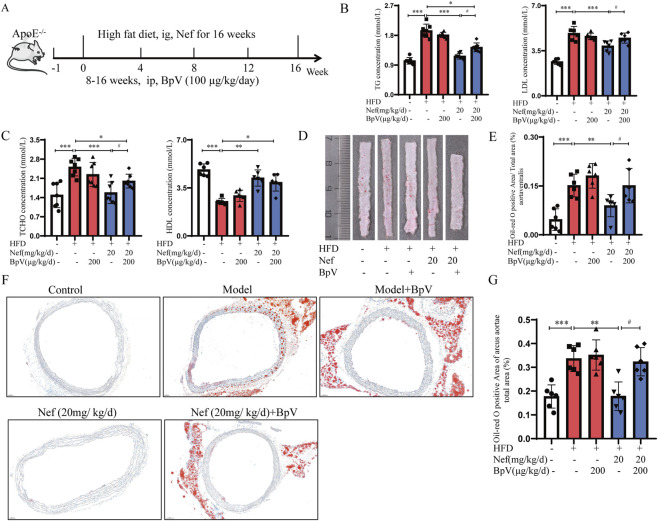
Nef significantly reduces AS plaque formation by targeting PTEN. **(A)** Schematic diagram of the animal experiment induced by HFD, intragastric administration of Nef, and intraperitoneal injection of BpV (n = 6). **(B,C)** Results of blood lipid biochemical tests (n = 6). **(D–G)** Results of Oil Red O staining of the aortic arch and aortaventralis (n = 6). *P < 0.05, **P < 0.01, ***P < 0.001 versus model group, ^#^ *P* < 0.05, ^##^ *P* < 0.01, ^###^ *P* < 0.001 versus Nef (20 mg/kg/day) group, scale column:100 μm.

As previously studied, PTEN serves as a potential target for regulating the proliferation and migration of VSMCs (Hsieh et al., 2025). Our research confirmed that PTEN inhibition significantly promoted the proliferation and migration of VSMCs, and that PTEN is a direct target of Nef (Figures 3, 4). To further elucidate the molecular mechanism through which Nef targets PTEN to regulate lipid metabolism in the AS model mice, we inhibited the biological function of PTEN by intraperitoneal injection of BpV and elucidated the pharmacological effect of Nef in targeting PTEN to regulate atherosclerotic plaque formation (Figure 6A). We observed that Nef significantly inhibited the HFD-induced increases in LDL, T-CHO, and TG levels, and the PTEN inhibitor BpV significantly weakened the lipid regulation effect of Nef (Figures 6B,C). Subsequently, atherosclerotic lesions in the aortic arch and aortaventralis were assessed using Oil Red O staining as in our previous study (Xie et al., 2025b). The model group revealed larger atherosclerotic lesion areas in the aortic arch and aortaventralis regions compared to the control mice. However, compared with the model group, Nef (20 mg/kg/day) treatment significantly alleviated atherosclerotic lesion areas in the aortaventralis and arcus aortae (Figures 6D–G). Moreover, competitive antagonism of PTEN significantly weakened the inhibitory effect of Nef on atherosclerotic plaque formation in the aortaventralis and arcus aortae (Figures 6D–G). Our study indicates that Nef targeting PTEN can inhibit atherosclerotic plaque formation, and that PTEN is a potential target for AS treatment.

## 4 Discussion

As the pathological basis of myocardial infarction, angina pectoris, and coronary heart disease, the treatment of AS significantly reduces atherosclerotic cardiovascular diseases (Wang et al., 2024; Falk, 2006). AS is an age-related disease. Vascular endothelial damage, increased oxidative stress levels, and,Ox-LDL promoted macrophage foaming are typical characteristics of AS, especially in elderly patients. Thus, inhibiting macrophage foam cell formation and abnormal VSMC proliferation and migration, as well as maintaining plaque stability, are recognized as effective strategies for treating AS (Miano et al., 2021; De Meyer et al., 2024). Our previous studies have demonstrated that Nef significantly inhibits macrophage foam formation and disrupts the glycolytic reprogramming induced by Ox-LDL (Xie et al., 2025b). Nevertheless, the pharmacological effects and molecular mechanisms of Nef on the VSMC proliferation and migration remain unclear, and its specific direct target has not yet been identified. In this study, we intragastrically administered Nef to HFD-induced ApoE^−/−^ mice and assessed atherosclerotic plaque formation. The results indicated that Nef inhibited atherosclerotic plaque formation and reduced serum levels of LDL, T-CHO, and TG induced by HFD (Figure 1). We observed that Nef significantly inhibited PCNA expression in the aortic arch, as determined by immunohistochemistry, indicating that Nef has good anti-AS pharmacological effects and can significantly inhibit VSMC proliferation. The increase in arterial intima thickness formed by abnormal proliferation and migration of VSMCs is generally considered an early lesion of AS (Back et al., 2019). Furthermore, our results found that Nef significantly inhibits the abnormal proliferation and migration of VSMCs induced by 3% FBS (Figure 2).

PTEN, as a tumor suppressor gene, can significantly inhibit the proliferation and invasion of tumor cells (Malaney et al., 2017). Meanwhile, PTEN can inhibit the PI3K/AKT and AKT/mTOR pathways, which regulate the proliferation and invasion of tumor cells (Chen et al., 2019). Moreover, PTEN deficiency promotes pathological vascular remodeling of human coronary arteries (Moulton et al., 2018), and inhibition of PTEN expression significantly promotes VSMC proliferation and migration, accelerating the formation and lesion of AS plaques (Zhu et al., 2019). In this study, we observed that Nef can target and bind to PTEN to form a Nef-PTEN complex (Figures 3A–C). CETSA and DARTs experiments confirmed that PTEN is a direct target of Nef and that CYS-130 and PHE152 are the direct binding checkpoints of Nef-PTEN (Figures 3E,F). More interestingly, Nef significantly enhanced the expression of PTEN mRNA and protein levels by qRT-PCR, Western blotting, and immunofluorescence (Figure 4), which is consistent with previous studies (Poornima et al., 2013). Moreover, we identified that inhibition of PTEN expression significantly inhibited the abnormal proliferation and migration of VSMCs and inhibited the regulatory effect of Nef on the abnormal proliferation and migration of VSMCs (Figures 4E,F).

**FIGURE 7:**
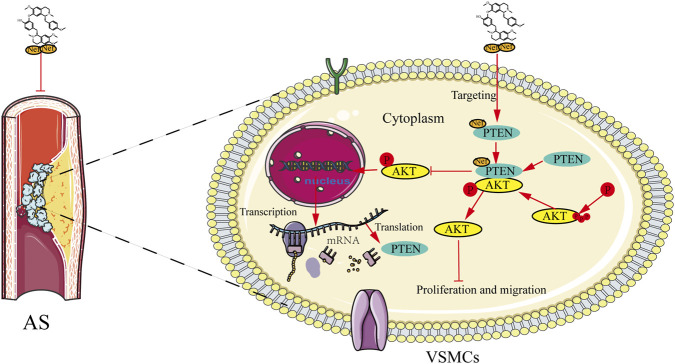
Neferine inhibits abnormal proliferation and migration of VSMCs in AS by targeting the PTEN/AKT complex.

Previous studies have revealed that inhibiting the AKT signaling pathway can significantly inhibit the abnormal proliferation and migration of VSMCs and alleviate atherosclerotic plaque formation (Xiao et al., 2023; Lin et al., 2024). In the present study, we found that Nef treatment did not significantly affect AKT mRNA or protein expression levels but significantly inhibited p-AKT expression (Figures 5A,B). Furthermore, we observed that PTEN and AKT formed PTEN-AKT complexes, and that Nef treatment significantly promoted the formation of PTEN-AKT complexes by molecular dynamics simulations and Co-IP experiments (Figures 5C–K). Notably, we found that competitive inhibition of PTEN significantly weakened AKT phosphorylation by Nef, and the regulation of the PTEN-AKT complex (Figures 5L,M). Meanwhile, competitive blockade of PTEN significantly inhibited the regulatory effect of Nef on the formation of atherosclerotic plaques in ApoE^−/−^ mice induced by HFD and promoted the formation of atherosclerotic plaques (Figure 6). Overall, PTEN is a direct target of Nef, and CYS130 and PHE252 are the binding sites of the PTEN-Nef complex. Nef targets PTEN to promote PTEN-AKT complex formation, thereby inhibiting AKT phosphorylation and VSMC proliferation and migration, suggesting that PTEN is a potential target for preventing and treating AS (Figure 7). More importantly, Nef is derived from medicinal and edible Chinese herbs, which has good safety and efficacy, providing research support for the clinical application of Nef in AS and for drug development. However, our study still has some limitations: (1) This study has not yet clarified the specific sites at which Nef targets PTEN to regulate AKT phosphorylation. (2) The crystal structure of the Nef-PTEN complex has not yet been resolved.

## Data Availability

The original contributions presented in the study are included in the article/supplementary material, further inquiries can be directed to the corresponding author.
